# Monitoring HPV Prevalence and Risk Cofactors for Abnormal Cytology in the Post-Vaccination Period among Croatian Women

**DOI:** 10.3390/v16040642

**Published:** 2024-04-20

**Authors:** Ena Pešut, Ivana Šimić, Rajko Fureš, Nina Milutin Gašperov, Cvjetko Lež, Fabijan Feratović, Tomica Kukina Žvigač, Magdalena Grce, Ivana Erceg Ivkošić, Ivan Sabol

**Affiliations:** 1 Division of Molecular Medicine, Ruđer Bošković Institute, Bijenička Cesta 54, 10000 Zagreb, Croatia; ena.pesut@irb.hr (E.P.); ivana.simic@irb.hr (I.Š.); nmilutin@irb.hr (N.M.G.); grce@irb.hr (M.G.); 2 General Hospital Zabok, Bračak 8, 49210 Zabok, Croatia; rajko.fures@bolnica-zabok.hr (R.F.); cvjetko.lez@bolnica-zabok.hr (C.L.); fabijanferat@gmail.com (F.F.); tkukina@gmail.com (T.K.Ž.); 3 Faculty of Dental Medicine and Health Osijek, Josip Juraj Strossmayer University Osijek, Crkvena 21, 31000 Osijek, Croatia; 4 Faculty of Health Studies, University of Rijeka, Viktora Cara Emina 5, 51000 Rijeka, Croatia; 5 Special Hospital Sveta Katarina, Branimirova 71 E, 10000 Zagreb, Croatia

**Keywords:** HPV, cervical cancer, prevalence, screening, vaccination

## Abstract

The incidence and mortality rate of cervical cancer in Croatia remains a health challenge despite screening efforts. Besides the persistent infection with HPV, the development of cancer is also associated with some cofactors. The goal of this study was to assess circulating HPV genotypes and risk factors for the development of cervical precancer after almost 16 years from the onset of HPV vaccination in Croatia. In this study, a total of 321 women attending gynecological care were evaluated. Relevant medical and demographic information, including cytology, were collected. HPV genotyping was performed by PCR. Comparing the HPV types found in circulation in the pre-vaccination (1999–2015) and post-vaccination periods (2020–2023), a statistically significant reduction in HPV 31 was noted, while the overall prevalence increased in the post-vaccination period. Besides the expected HPV positivity as a risk factor, the history of smoking was associated with LSIL or worse cytology at enrollment. For the first time, this population study revealed a statistically significant shift in the HPV genotype in the post-vaccination period, as well as the confirmation of risk factors for the development of abnormal cytology among Croatian women.

## 1. Introduction

Cervical cancer (CC) is still among the most common cancers in women and remains one of the major global health challenges, although it should not be [1]. According to the latest data, around 600,000 women in the world get CC every year, and the highest number of new cases was recorded in low- and middle-income countries [2,3]. In Croatia, the incidence is still relatively high, with 276 cases annually (ASR [age-standardized (EU) rate] 11.0/100,000), as is the mortality (ASR 4.2/100,000) [4].

Persistent infection with the sexually transmitted human papillomavirus (HPV) is responsible for 90–100% of CC cases in women [3]. To prevent the infection and thus indirectly reduce cancer incidence, several vaccines became available in the last decades, with worldwide distribution starting in 2006 [5]. The efforts to eliminate the burdens of CC were also recently strengthened in 2020 by the WHO global strategy, which aims for the elimination of CC [6]. To accelerate elimination efforts, countries should reach 90% of HPV-vaccinated girls at the age of 15, have 70% of women examined with a screening cytology test by the age of 35 and again by the age of 45, and have 90% of women with identified CC receiving treatment by 2030 [6]. Up to March 2022, 117 countries (60% of WHO member countries, approximately one-third of the global target population) already included the HPV vaccine in their routine national vaccination schedule [7]. In Croatia, preliminary vaccination campaigns with the bivalent (Cervarix^®^) and the quadrivalent (Gardasil 4^®^) HPV vaccine focusing on the Zagreb region started in 2007 (voluntary and freely available to 8th graders, 14–15 years old), while countrywide efforts started in 2016 offering the nine-valent (Gardasil 9^®^) HPV vaccine [8] (included in the national immunization program, voluntary and free for 8th graders throughout the country). Since 2019, free catch-up vaccination has been offered for people younger than 26, depending on vaccine availability. According to recent Croatian data, the number of vaccinated young people under the age of 25 has increased manifold since 2016, when 5282 young people received the first dose, to 21,306 vaccinated with the first dose in 2022 [9]. However, the number of recorded vaccinated people against HPV remains low considering the target population [10]. Most recent representative national survey data suggests that approximately 18.3% of young adults aged 18–25 years have been vaccinated against HPV, of which 65.6% were women [11]. 

Our previous work published in 2017, before the global strategy, presented a comprehensive and extensive study on the distribution of the most common HPV types among Croatian women in order to better predict and monitor the impact of HPV vaccination and further design effective preventive program strategies in Croatia [12]. Given the low vaccination rates, as well as worldwide trends that contribute to vaccine hesitancy [13], the goal of this study was to assess the current impacts of vaccination on the HPV type prevalence after almost 16 years from the accessibility of vaccination and 8 years after the implementation of countrywide free vaccination. 

Moreover, besides the presence of HPV, it is known that the development of CC is also associated with some cofactors, such as smoking, use of oral contraceptives, high parity, number of sexual partners, age at first intercourse, and co-infection with other sexually-transmitted diseases [14,15]. Of those, indirect HPV exposure measures, like age at first intercourse and number of partners, as well as smoking, were also relevant for developing high-grade abnormalities in younger women [16]. Hence, the secondary goal of this study was to assess the HPV types circulating in the population as well as the distribution, prevalence, and genotypes found within the cytology-tested women and analyze potential risk factors for the development of cervical cancer precursor lesions. 

## 2. Materials and Methods

Study population consisted of women attending routine gynecology examinations or referred (opportunistic screening) to the Special Hospital Sveta Katarina, within the capital city of Zagreb or General Hospital Zabok that services wider Zagreb County area. The patients were enrolled from June 2020 to December 2023. All women were informed about the goals of the project and signed the informed consent to participate in the study. The first cervical cytological samples (classic Pap smear) were taken for routine cytological diagnosis and hospital patient management. For this study, a second cytology sample was obtained from consenting women with a Cervex-brush^®^ (Rovers Medical Devices, Oss, The Netherlands) in liquid-based cytology (LBC) solution NOVAprep^®^ OrangeHQ+ (Novacyt, Velizy-Villacoublay, France). Besides collecting the cervical samples, the basic clinical and demographic parameters were recorded. The women were asked about their smoking history, previous live births, previous abortions, family history of cancer, and the use of non-prescription medication, as well as prescription medication, during the examination, including HPV vaccination history. Relevant medical information, including the cytology results of the concurrent routine swab, was collected from the hospital information system for each patient. Cytology results, performed according to each hospital routine procedure, were classified according to the Bethesda classification. For purposes of analysis, cytology results were grouped into normal, atypical squamous or glandular cells of unknown significance (ASC/AGC-US; there was only a single AGC-US case that was grouped here for simplicity), low-grade squamous intraepithelial lesion (LSIL) and high-grade squamous intraepithelial lesion (HSIL) cases. Only adult women above 18 years of age were enrolled. Patients who underwent surgical management of precancerous cervical lesions within the previous 5 years were subsequently excluded from the analysis. 

DNA was isolated from an aliquot of 1 mL of the resuspended sample using the QIAamp DNA Kit (Qiagen, Hilden, Germany). The total DNA concentration and purity were measured using a NanoPhotometer (Implen, Munich, Germany). Polymerase chain reaction (PCR) for HPV was performed with consensus HPV-primers PGMY, GP5+/GP6+, and LC, while genotyping was done with type-specific primers for HPV 6/11, HPV 16, HPV 18, HPV 31, HPV 33, HPV 45, HPV 52, and HPV 58, as previously described [17]. Beta-globin PCR amplification was used as an internal control. Separate laboratory areas were used for DNA isolation, PCR, and post-amplification processing. PCR reactions were set up in a laminar flow PCR cabinet decontaminated with UV irradiation to further limit likelihood of contamination. Samples were considered to be HPV-positive if one of the consensus or type-specific PCRs were positive. Samples PCR-positive for HPV 6/11 were considered to be low-risk HPV (LR-HPV) unless concurrently positive for either of the other tested high-risk HPV types (HR-HPV; i.e., HPV 16, 18, 31, 33, 45, 52, and 58). Samples that were positive with consensus primers directed PCR and negative using type-specific primers were considered non-vaccine HPV types (i.e., none of the vaccine types HPV 6/11, 16, 18, 31, 33, 45, 52, or 58).

Data were collected in Excel tables, and statistical analysis was performed using MedCalc (v20.11, MedCalc Software bv, Ostend, Belgium). Categorical variables were summarized with percentages and assessed with Chi-square (χ^2^) test. Age, as the only continuous variable, was found to be of abnormal distribution by Kolmogorov–Smirnov test. Age groups were made in consideration of expected age of participants when the vaccine was introduced in Croatia. Participants under the age of 30 would have been the target of the initial vaccination rollout in 2007, which targeted children attending the final year of elementary school. *p*-value < 0.05 was considered statistically significant.

## 3. Results

In this study, a total of 321 women aged 18–77 years from the opportunistic screening population attending gynecological care were evaluated (Supplementary Table S1). Most women (50.2%) were between 31–45 years of age, and the majority had normal cytology (63.2%).

Table 1 summarizes the demographic and medical characteristics of women with or without LSIL or worse cytology at enrollment. The detailed prevalence of potential risk factors according to the hospital is shown in (Supplementary Table S1). As expected, HPV positivity was strongly associated with cervical lesions (*p* < 0.0001). The history of smoking was also associated with LSIL or worse cytology (*p* = 0.0357), as was the number of cigarettes per day (*p* = 0.0004). Other possible risk factors (parity, abortions, or previous cancer history) had no statistically significant association with LSIL or worse cytology abnormalities.

The distribution of the individual HPV types in the patients with or without LSIL or worse cytology lesions is shown in Table 2, while a detailed distribution is shown in Supplementary Table S2. As expected, LSIL or worse cases had more HR-HPV infections, as well as HPV of any type (Table 2).

**Figure 1:**
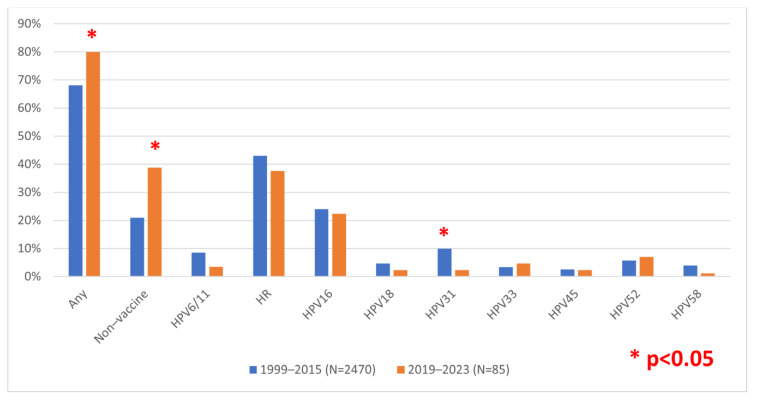
Comparison of HPV prevalence in low-grade squamous intraepithelial lesion (LSIL) or worse cases during the pre- or post-vaccination period in Croatia. Non-vaccine types, PCR positive with consensus but negative with type-specific primers (i.e., none of the HPV types 6/11, 16, 18, 31, 33, 45, 52, or 58).

Furthermore, we aimed to compare the currently circulating HPV types in a post-vaccination setting with the HPV types circulating in the population before the introduction of vaccination at the national level based on our previous results on samples collected between 1999 and 2015 [12]. Due to differences in sample collection and the awareness of HPV detection benefits, which was historically mostly performed in cases of atypia, only LSIL or worse cytology findings were considered for this inter-study comparison (Supplementary Table S2). Comparing the HR-HPV types found in circulation in the pre-vaccination and post-vaccination periods, a statistically significant reduction of HPV 31 (*p* = 0.02) was noted, as well as a slight reduction without statistical significance in HPV types 16 (*p* = 0.731), 18 (*p* = 0.318), 45 (*p* = 0.892), and 58 (*p* = 0.186) (Figure 1). However, HPV types 33 and 52 were found slightly more often, again without reaching statistical significance (*p* = 0.501 and *p* = 0.611, respectively). Furthermore, there was a statistically significant increase in non-vaccine HPV types despite the fact that the HPV PCR detection methods remained the same between studies. The overall prevalence also increased in the recent period (80% vs. 68.1%, *p* = 0.02). 

To further investigate potential vaccine effects, we selected only women in the youngest age group (between 18 and 30 years of age), which could have recently benefited from vaccination efforts from both cohorts. The results remained comparable with an overall decrease in HR-HPV types, as well as decreases in HPV16, 31, and 58, in particular (Supplementary Figure S1). Overall, increases in any type and non-vaccine types were also notable. However, due to the limited number of cases, only the increase in non-vaccine types in the post-vaccination cohort reached statistical significance for younger women (*p* < 0.0001).

## 4. Discussion

Cervical cancer (CC) is a major public health problem that can be attributed to certain HPV types in almost 100% of cases [18]. In Croatia, vaccination is currently carried out with the nine-valent HPV vaccine (Gardasil 9) [9], which covers the nine most common HPV types (HPV 6, 11, 16, 18, 31, 33, 45, 52, and 58) and therefore has the potential to provide protection for about 90% of CC cases and genital warts [5]. 

In addition to our previous studies [12,19,20], the latest studies on the prevalence of HPV in the pre-vaccination period in Croatia were evaluated by Kaliterna et al. in Southern Croatia, which also showed higher overall HPV prevalence as well as that the most common type was HPV 16 [21]. Even though many years have passed since the introduction of the HPV vaccine in Croatia, to our knowledge, this study was the first to assess the trends in the HPV prevalence in the post-vaccination period compared to the pre-vaccination situation in Croatia.

The present study involved a total of 321 women who came for a regular gynecological examination in two hospitals (Zagreb and Zagreb County area), and we applied the same HPV PCR detection methods as for the pre-vaccination period. In the pre-vaccination setting, the awareness about HPV testing was lower, and HPV testing was mostly performed for women with abnormal cytology. This changed somewhat in the subsequent years, and the new cohort included a more general screening population yet not a completely strictly screening population. Thus, to allow for a more meaningful comparison, we compared only the prevalence of HPV in LSIL or worse cytological findings from both cohorts. We determined that there was no statistically significant change in the overall prevalence of HR-HPV compared to the period before (1999–2015) [12] and after vaccination (2020–2023). However, we observed that there was a statistically significant decrease in the prevalence of HPV 31 and an increase in the number of the non-vaccine types (none of the nine most common types targeted by PCR). This could be due to direct effects on the vaccine-targeted types, as also observed in the other populations listed below. However, there was no statistically significant change in the prevalence of HPV 16 in the post-vaccination period. Since we found HPV 16 somewhat more often (9.6%) than what was recently reported for the predominantly asymptomatic general screening population in Southern Croatia (3.6%) [22], the lack of expected HPV 16 decrease could also be due to the population cohort differences either in the pre- or post-vaccination period we studied.

Our study is comparable with the study conducted in Spain, which included the pre- and post-vaccination period on women who came for regular gynecological examinations regardless of whether they had been vaccinated [23]. Through follow-up, Freire-Salinas et al. determined the replacement of genotypes that were not included in the vaccine (Cervarix^®^ and Gardasil 4^®^ in their case) [23]. They found a decrease in the genotypes HPV 6/11 (statistically significant) and HPV 16 (not statistically significant) and a statistical increase in the types HPV 31, HPV 52, and HPV 45 (not included in the vaccine used) [23]. This is similar to our study, where non-vaccine HPV types increased and where only some vaccine types decreased significantly. 

While our study did not focus on HPV-vaccinated versus unvaccinated women since the vaccination coverage is low, it needs to be emphasized that several studies conducted within the decade of vaccination implementation already showed changes in the HPV types circulating within those populations. The study by Markowitz et al. in the United States showed that within 9–10 years of vaccine introduction, the vaccine-type HPV prevalence decreased and that there were declines in both vaccinated and, more importantly, unvaccinated women, showing the evidence of direct and indirect (herd) protection [5]. Garcia et al. [24] recently reported that even 5 years after the HPV vaccination started in Sweden, a reduction in vaccine-covered types could be seen in vaccinated individuals. Interestingly, vaccinated women below 31 years of age had no cases of infections with vaccine-covered types (6/11/16/18); however, HPV-positive vaccinated women with dysplasia had more infections with non-vaccine-covered types, which suggests some level of type switching. Also, in Sweden, 10 years after the vaccination started, the prevalence of HPV 16 significantly decreased in vaccinated as well as non-vaccinated women compared to the pre-vaccination cohort [25]. Another similarity was the increase in HPV 52 as well as increases in HPV types not included in the vaccine. In one study, a significant reduction of HPV was seen after only 4 years of vaccination onset in unvaccinated women [26]. The observed changes to non-vaccine types are further corroborated by a large study in the Finnish population looking at community-level genotype diversity 4 and 8 years after vaccine implementation [27]. Therein, the authors noted that, on the community level, the ecological diversity of types increased from 4 to 8 years post-vaccination, probably by ecological niche occupation by the non-targeted types. They also noted the increase in HPV52. Again, in contrast to Croatia, a significant decrease in vaccine-targeted types could already be seen 4 years after vaccination.

There are also similarities with the large meta-analysis by Sabeen and Ravishan (covering Australia, Europe, Asia, and the United States) in the post-vaccination period, which explicitly included vaccinated and unvaccinated female populations. They concluded that there was a significant reduction in the overall prevalence of vaccine-derived HPV types among young, vaccinated women [28]. On the other hand, one Norwegian study reported significant reductions for unvaccinated types [29]. The Italian study reported a higher prevalence of the non-vaccine HPV type, HPV 42, in the post-vaccination period, but still, there was no genotype replacement at a statistically significant level [30].

There can be different explanations for the observed changes in the differences in the HPV types circulating in pre- and post-vaccination settings (or vaccinated and non-vaccinated cohorts) where vaccinated women and populations show a decrease in vaccine-covered types but an increase in non-vaccine HR-HPV types in the lesions [31]. For example, type replacement/selection pressure [27], where vaccine-targeted types are depleted, and the niche is filled by non-targeted types or unmasking [32], where untargeted types are present but cannot be detected due to technical limitations until vaccine-targeted types are depleted in a population. Additional changes in screening programs, sampling, sexual behaviors, patient awareness, or other population characteristics could affect the intra-study comparison results. Only some of the above were reliably addressed in our study, i.e., using the same low multiplex PCR methodology addresses the technical aspects, while no implementation of a more organized screening program decreases the impact of potential increased patient awareness or enhanced surveillance. Unfortunately, our post-vaccination period is not much different from prior opportunistic screening used in the pre-vaccination period. While the differences in sexual behaviors could not be assessed, the most likely explanation for the observed HPV genotype changes would be the type replacement hypothesis. However, the lack of HPV 16 depletion indicates that further efforts are needed.

The end goal of vaccination against HPV is certainly a reduction in cancer cases. Australia is one of the leading countries in this fight, where the introduction of HPV vaccination led to major changes in HPV prevalence as well as cervical abnormalities [33]. Australia also started the vaccination efforts in 2007, as did Croatia tentatively; however, in Australia, already in 2010–2012, post-vaccination showed a drastic reduction in HPV 16 along with a halving of HSIL abnormalities in the youngest age group. However, in Croatia, even 10 years later, we observe no significant changes in HPV 16, likely because of the relatively low number of vaccinated women. Another very recent study published by the Scottish Cancer Registry evaluating 450,000 women noted the fully vaccinated cohort exhibited no invasive cancer cases compared to the rate of 8.4/100,000 in unvaccinated women [34], further highlighting the large health benefits of successfully implemented vaccination program towards which Croatia is aiming. 

Some other European countries already had a very high vaccination response (over 70%) by 2017 in certain age groups, for example, Iceland, the United Kingdom, Norway, Spain, and Sweden [35,36]. Some countries geographically close to Croatia achieved a significantly higher vaccination rate by 2017, such as Slovenia (46% target age 11–12 years), the Czech Republic (58% target age 13 years), and Italy (62% target age 11 years) [35,36]. Herein, the most common determinants of HPV vaccine hesitancy identified in European countries include the quality and quantity of available information about the HPV vaccine and its safety and, unfortunately, the lack of trust in health authorities [37]. Therefore, the most recent study in Croatia determined that the odds of vaccination hesitancy were significantly higher among those who were more religious but lower among women, participants who reported a higher perceived risk of sexually transmitted infections, and those who recognized that HPV could result in CC [11].

Our secondary objective in this study was to confirm the associations of the recognized risk factors with abnormal cytology [15]. Although we were unable to collect sufficient data on some risk factors, such as the use of oral contraceptives [38], because only a small number of women reported this information, we nevertheless show that there is a connection between smoking as well as the number of cigarettes per day, with the development of LSIL or worse cytological lesions in the Croatian population. A large meta-analysis of various countries by Berrington de González et al. also showed that smoking is a risk for the development of cervical cancer [39]. Another study from Ireland showed that the number of cigarettes smoked is associated with a dose-dependent increased risk of CIN 2/3 among women who have mild abnormal cervical smears, which could be more comparable to our study [40].

## 5. Conclusions

This post-vaccination population study on the prevalence and distribution of HPV genotypes in the general female population in Zagreb and Zagreb County area will be valuable for monitoring the trend of HPV in Croatia after the start of vaccination. Despite the much-delayed implementation of full-scale vaccination efforts in Croatia, some tentative changes in individual HPV-type prevalences can be seen, with the reduction in HPV 31 being the most relevant. However, since HPV 16 prevalence remained stable, further efforts in increasing vaccination coverage in Croatia are needed before decreases in cytological abnormalities become as significant as in other Western populations.

## Figures and Tables

**Table 1:** Demographic and medical characteristics of women with or without abnormal cytology.

Variable		Normal/ASCUS (N = 236) N (%)	LSIL+ (N = 85) N (%)	*p*-Value
**Age groups**	18–30	67 (28.4)	29 (34.1)	0.2580
	31–45	117 (49.6)	44 (51.8)	
	46+	52 (22)	12 (14.1)	
**Smoking history**	Non-smoker	175 (74.2)	52 (61.2)	0.0357
	Smoker	60 (25.4)	31 (36.5)	
	Not specified	1 (0.4)	2 (2.3)	
**Number of** **cigarettes per day**	Not reported	3 (1.3)	9 (10.6)	0.0004
	Non-smoker	175 (74.1)	52 (61.2)	
	1–5/day	18 (7.6)	5 (5.9)	
	6–10/day	16 (6.8)	12 (14.1)	
	11–20/day	24 (10.2)	7 (8.2)	
**Parity**	Nulliparous	93 (39.4)	34 (40)	0.4861
	1–2	119 (50.4)	46 (54.1)	
	3+	24 (10.2)	5 (5.9)	
**Abortions**	Yes	203 (86.0)	68 (80.0)	0.1903
	No	33 (14.0)	17 (20.0)	
**Cancer history**	Nothing reported	140 (59.3)	57 (67.1)	0.2098
	Family or personal history	96 (40.7)	28 (32.9)	
**HPV positivity ^1^**	Negative	157 (66.5)	17 (20.0)	<0.0001
	Positive	79 (33.5)	68 (80.0)	
	LR-HPV	2 (0.8)	3 (3.5)	
	Non-vaccine types	38 (16.1)	33 (38.8)	
	HR-HPV	39 (16.5)	32 (37.6)	

^1^ HR-HPV types 16, 18, 31, 33, 45, 52, and 58; LR-HPV types 6/11; non-vaccine types, PCR positive with consensus but negative with type-specific primers (i.e., none of HPV types 6/11, 16, 18, 31, 33, 45, 52, or 58).

**Table 2:** Prevalence of HPV types among cytological categories in study population.

HPV Positivity ^1^	Normal (N = 236) N (%)	LSIL+ (N = 85) N (%)	Total (N = 321) N (%)
**Negative**	157 (66.5)	17 (20.0)	174 (54.2)
**Any HPV**	79 (33.5)	68 (80.0)	147 (45.8)
**Non-vaccine types**	38 (16.1)	33 (38.8)	71 (22.1)
**LR-HPV (HPV 6/11)**	3 (1.3)	3 (3.5)	6 (1.9)
**HR-HPV**	39 (16.5)	32 (37.6)	71 (22.1)
**HPV 16**	21 (8.9)	19 (22.4)	40 (12.5)
**HPV 18**	2 (0.8)	2 (2.4)	4 (1.2)
**HPV 31**	5 (2.1)	2 (2.4)	7 (2.2)
**HPV 33**	2 (0.8)	4 (4.7)	6 (1.9)
**HPV 45**	5 (2.1)	2 (2.4)	7 (2.2)
**HPV 52**	8 (3.4)	6 (7.1)	14 (4.4)
**HPV 58**	2 (0.8)	1 (1.2)	3 (0.9)

^1^ HR-HPV types 16, 18, 31, 33, 45, 52, and 58; LR-HPV types 6/11; non-vaccine types, PCR positive with consensus but negative with type-specific primers (i.e., none of HPV types 6/11, 16, 18, 31, 33, 45, 52, or 58). Column totals are above 100% due to multiple infections.

## Data Availability

The data that support the findings of this study are contained within the manuscript and Supplementary Material. Further data are available from the corresponding author upon reasonable request.

## Supplementary Materials

The following supporting information can be downloaded at: https://www.mdpi.com/article/10.3390/v16040642/s1, Supplementary Table S1. Demographic information of the study population (N = 321); Supplementary Table S2. Comparison of HPV types in pre- (1999–2015) and post-vaccination periods (2020–2023) across different cytology result groups. Supplementary Figure S1. Comparison of HPV prevalence in low-grade squamous intraepithelial lesion (LSIL) or worse cases during the pre- or post-vaccination period in Croatia in young women (18–30 years). Non-vaccine types, PCR positive with consensus but negative with type-specific primers (i.e., none of HPV types 6/11, 16, 18, 31, 33, 45, 52, or 58).
