# Supplementary material for: Monitoring HPV Prevalence and Risk Cofactors for Abnormal Cytology in the Post-Vaccination Period among Croatian Women
Source: Viruses. 2024 Apr 20;16(4):642. doi: 10.3390/v16040642 (PMC11054414; doi:10.3390/v16040642)
Supplement: Supplementary file 1 [file viruses-16-00642-s001.zip › viruses-2929910-supplementary.pdf]

Supplementary Table S1. Demographic information of the study population (N = 321)

Variable		General Hospital Zabok (N=170)	Special Hospital Sveta Katarina (N=151)	Total (N=321)
Patient age	Median (IQR)[1]	39 (29-48)	34 (29-39)	37 (29-43)
	Mean ± SD[2]	40 ± 13.1	34.3 ± 7.8	37.3 ± 11.3
	Range (Min-Max)	19 - 77	18 - 64	18 - 77
Age groups	18-30	49 (28.8)	47 (31.1)	96 (29.9)
	31-45	67 (39.4)	94 (62.3)	161 (50.2)
	46+	54 (31.8)	10 (6.6)	64 (19.9)
Smoking history	Non smoker	122 (71.8)	105 (69.5)	227 (70.7)
	Smoker	38 (22.4)	44 (29.1)	82 (25.5)
	1-5 /day	8 (4.7)	15 (9.9)	23 (7.2)
	6-10 /day	19 (11.2)	9 (6)	28 (8.7)
	11-20 /day	11 (6.5)	20 (13.2)	31 (9.7)
	not reported	10 (5.9)	2 (1.3)	12 (3.7)
Parity	Nuliparous	51 (30)	76 (50.3)	127 (39.6)
	1-2	94 (55.3)	71 (47)	165 (51.4)
	3+	25 (14.7)	4 (2.6)	29 (9)
History of abortion		27 (15.9)	23 (15.2)	50 (15.6)
Cancer history[3]		47 (27.6)	77 (51)	124 (38.6)
Cytology	Normal	72 (42.4)	131 (86.8)	203 (63.2)
	Abnormal	98 (57.6)	20 (13.2)	118 (36.8)
	ASC/AGC-US	29 (17.1)	4 (2.6)	33 (10.3)
	LSIL	37 (21.8)	11 (7.3)	48 (15)
	HSIL	32 (18.8)	5 (3.3)	37 (11.5)

[1]IQR-interquartile range, age data was not following normal distribution; [2]SD-standard deviation; [3]personal or family history of cancer

Supplementary Table S2. Comparison of HPV types in pre- (1999–2015) and post- vaccination periods (2020–2023) across different cytology result groups.

	**Normal**		**ASCUS**		**LSIL**		**HSIL**		**Total**	
	**Pre**	**Post**	**Pre**	**Post**	**Pre**	**Post**	**Pre**	**Post**	**Pre**	**Post**
**Total N**	**72**	**203**	**1029**	**33**	**1310**	**48**	**1246**	**37**	**3657**	**321**
Any	15 (20.8)	67 (33)	517 (50.2)	12 (36.4)	745 (56.9)	37 (77.1)	980 (78.7)	31 (83.8)	2257 (61.7)	147 (45.8)
X	6 (8.3)	31 (15.3)	156 (15.2)	7 (21.2)	285 (21.8)	23 (47.9)	243 (19.5)	10 (27)	690 (18.9)	71 (22.1)
6/11	1 (1.4)	3 (1.5)	72 (7)	0 (0)	111 (8.5)	1 (2.1)	101 (8.1)	2 (5.4)	285 (7.8)	6 (1.9)
HR	8 (11.1)	34 (16.7)	322 (31.3)	5 (15.2)	390 (29.8)	13 (27.1)	692 (55.5)	19 (51.4)	1412 (38.6)	71 (22.1)
16	5 (6.9)	18 (8.9)	159 (15.5)	3 (9.1)	188 (14.4)	7 (14.6)	415 (33.3)	12 (32.4)	767 (21)	40 (12.5)
18	0 (0)	2 (1)	32 (3.1)	0 (0)	47 (3.6)	1 (2.1)	71 (5.7)	1 (2.7)	150 (4.1)	4 (1.2)
31	1 (1.4)	4 (2)	90 (8.7)	1 (3)	83 (6.3)	0 (0)	166 (13.3)	2 (5.4)	340 (9.3)	7 (2.2)
33	0 (0)	1 (0.5)	41 (4)	1 (3)	29 (2.2)	0 (0)	54 (4.3)	4 (10.8)	124 (3.4)	6 (1.9)
45	1 (1.4)	4 (2)	14 (1.4)	1 (3)	27 (2.1)	2 (4.2)	38 (3)	0 (0)	80 (2.2)	7 (2.2)
52	2 (0)	6 (1)	51 (5)	2 (6.1)	61 (4.7)	3 (6.3)	85 (6.8)	3 (8.1)	199 (5.4)	14 (4.4)
58	0 (0)	2 (1)	30 (2.9)	0 (0)	40 (3.1)	1 (2.1)	60 (4.8)	0 (0)	130 (3.6)	3 (0.9)

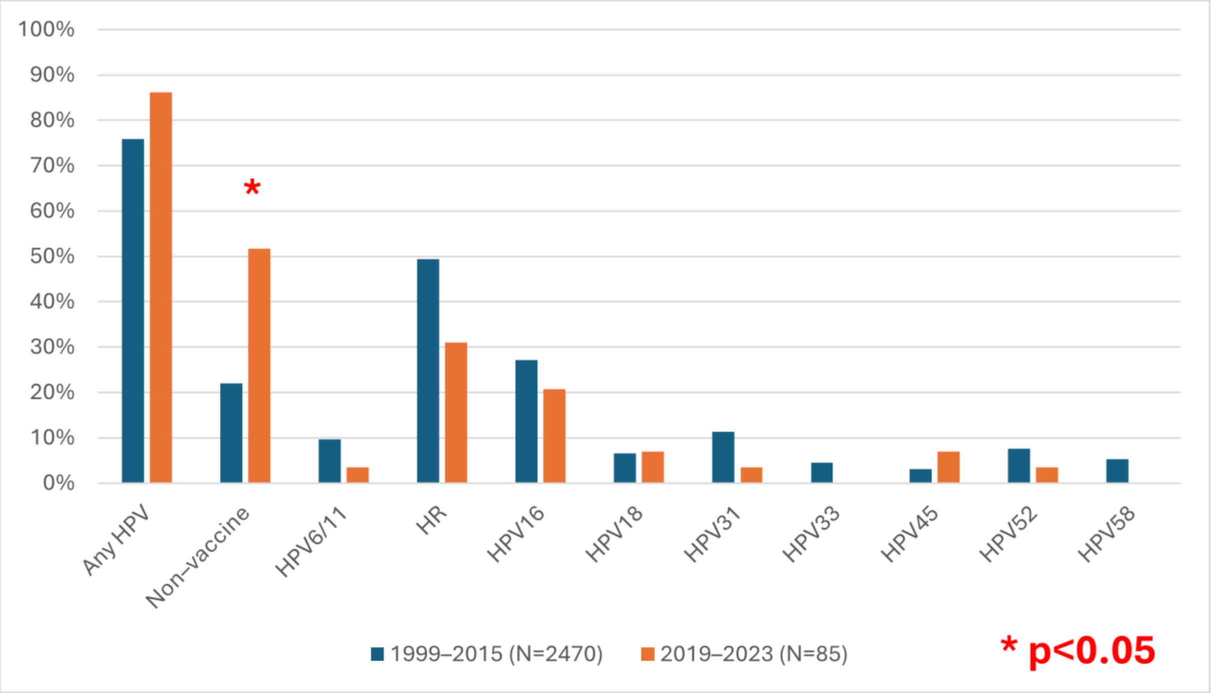

Supplementary Figure S1. Comparison of HPV prevalence in low-grade squamous intraepithelial lesion (LSIL) or worse cases during the pre- or post-vaccination period in Croatia in young women (18–30 years). Non-vaccine types, PCR positive with consensus but negative with type-specific primers (i.e., none of HPV types 6/11, 16, 18, 31, 33, 45, 52, or 58).
